# Somatosensory function in patients with secondary adrenal insufficiency treated with two different doses of hydrocortisone—Results from a randomized controlled trial

**DOI:** 10.1371/journal.pone.0180326

**Published:** 2017-07-07

**Authors:** Jorien Werumeus Buning, Karl-Heinz Konopka, Pauline Brummelman, Janneke Koerts, Robin P. F. Dullaart, Gerrit van den Berg, Melanie M. van der Klauw, Oliver Tucha, Bruce H. R. Wolffenbuttel, André P. van Beek

**Affiliations:** 1 Department of Endocrinology, University of Groningen, University Medical Center Groningen, Groningen, The Netherlands; 2 Drug Discovery Science & Management-Europe, Astellas Pharma Europe B.V., Leiden, The Netherlands; 3 Department of Clinical and Developmental Neuropsychology, University of Groningen, Groningen, The Netherlands; Medizinische Universitat Graz, AUSTRIA

## Abstract

**Background:**

Low cortisol levels are associated with several functional pain syndromes. In patients with secondary adrenal insufficiency (SAI), the lack in endogenous cortisol production is substituted by the administration of oral hydrocortisone (HC). Our previous study showed that a lower dose of HC led to an increase in reported subjective pain symptoms. Whether different doses of HC substitution alter somatosensory functioning in SAI patients has not been established yet.

**Methods:**

In this randomized double blind cross-over trial, forty-six patients with SAI participated. Patients randomly received either first a lower dose (0.2–0.3 mg HC/kg body weight/day) for 10 weeks followed by a higher dose (0.4–0.6 mg HC/kg body weight/day) for another 10 weeks, or vice versa. After each treatment period, blood samples were drawn and somatosensory functioning was assessed by determining the mechanical detection threshold (MDT), mechanical pain threshold (MPT), mechanical pain sensitivity (MPS) and the pain pressure threshold (PPT), according to the Quantitative Sensory Testing (QST) battery by the German Network on Neuropathic Pain.

**Results:**

The administration of the higher dose of HC resulted in significantly higher levels of cortisol (mean [SD] 748 [245] nmol/L) than the lower dose (537 [250] nmol/L, P<0.001). No differences were found in MDT, MPT, MPS and PPT z-scores between the two doses of HC. Furthermore, the number of patients showing sensory abnormalities did not differ between the two different doses.

**Conclusions:**

The results suggest that the dose of HC has no impact on somatosensory functioning in response to mechanical stimuli in patients with SAI, despite previously found altered subjective pain reports.

## Introduction

Stress hormones are known to play an important role in stress-related bodily disorders, such as functional pain syndromes. Stress, i.e. internal or external threats to the maintenance of homeostasis, activates the hypothalamic-pituitary-adrenal (HPA) axis in order to reestablish homeostasis. Activation of the HPA axis results among others in the secretion of cortisol. There is some evidence that cortisol increases the tolerance of pain [1,2]. Several functional pain syndromes are linked to hypocortisolism [1,3]. For instance, patients with fibromyalgia showed reduced 24 h-urinary cortisol excretion compared to healthy controls [4–6]. Fibromyalgia is characterized by central sensitization, a process of hypersensitivity of neural nociceptive pathways [7]. Cortisol has immunosuppressive effects, and reduced cortisol levels might disinhibit the secretion of inflammatory mediators and thereby promote the sensitization of peripheral or central nociceptive neurons [8]. On the other hand, it was found that higher cortisol levels are associated with decreased pain thresholds [9] and increased pain sensitivity in response to thermal stimuli [10]. Other studies were unable to find an effect of cortisol levels and pain sensitivity [11]. Thus, the relationship between cortisol levels and pain sensitivity remains unclear.

The relationship between cortisol levels and sensory functioning using mechanical stimuli has been studied in healthy volunteers [12]. However, in healthy individuals the negative feedback mechanisms of the HPA axis makes it difficult to study the relationship between cortisol levels and sensory functioning. Patients with secondary adrenal insufficiency (SAI) are characterized by a loss of endogenous cortisol production due to impaired hypothalamus or pituitary functioning, thereby lacking this negative feedback mechanism. These patients receive substitution therapy, usually oral tablets of hydrocortisone (HC). Because the negative feedback mechanisms is absent in patient with SAI, cortisol levels can be controlled externally, making them a unique group of patients to study the direct relationship between cortisol concentrations and somatosensory function.

As shown previously by our group, patients with SAI reported more pain symptoms as assessed by daily diaries during treatment with a lower dose of HC (total daily doses ranging from 15–20 mg HC) when compared to treatment with a higher dose of HC (total daily doses ranging from 30–40 mg HC) [13]. However, it is unknown whether these two substitution doses also influence somatosensory function. This study is part of a recently conducted randomized double blind cross-over study which assessed the effect of two different doses of HC on cognition [13], health related quality of life [14], blood pressure [15], pharmacokinetics and somatosensory functioning. Sensory function was assessed using parts of the German Research Network on Neuropathic Pain (DFNS) Quantitative Sensory Testing (QST) battery which assesses the perceptual response to systematically applied and quantifiable sensory stimuli [16,17]. QST has been shown to be valid, reliable and sensitive in quantifying sensory abnormalities [18–20]. It is hypothesized that patients show increased sensory functioning as assessed by mechanical stimuli after receiving a low dose of HC in comparison to after receiving a high dose of HC.

## Materials and methods

### Patients

**Fig 1:**
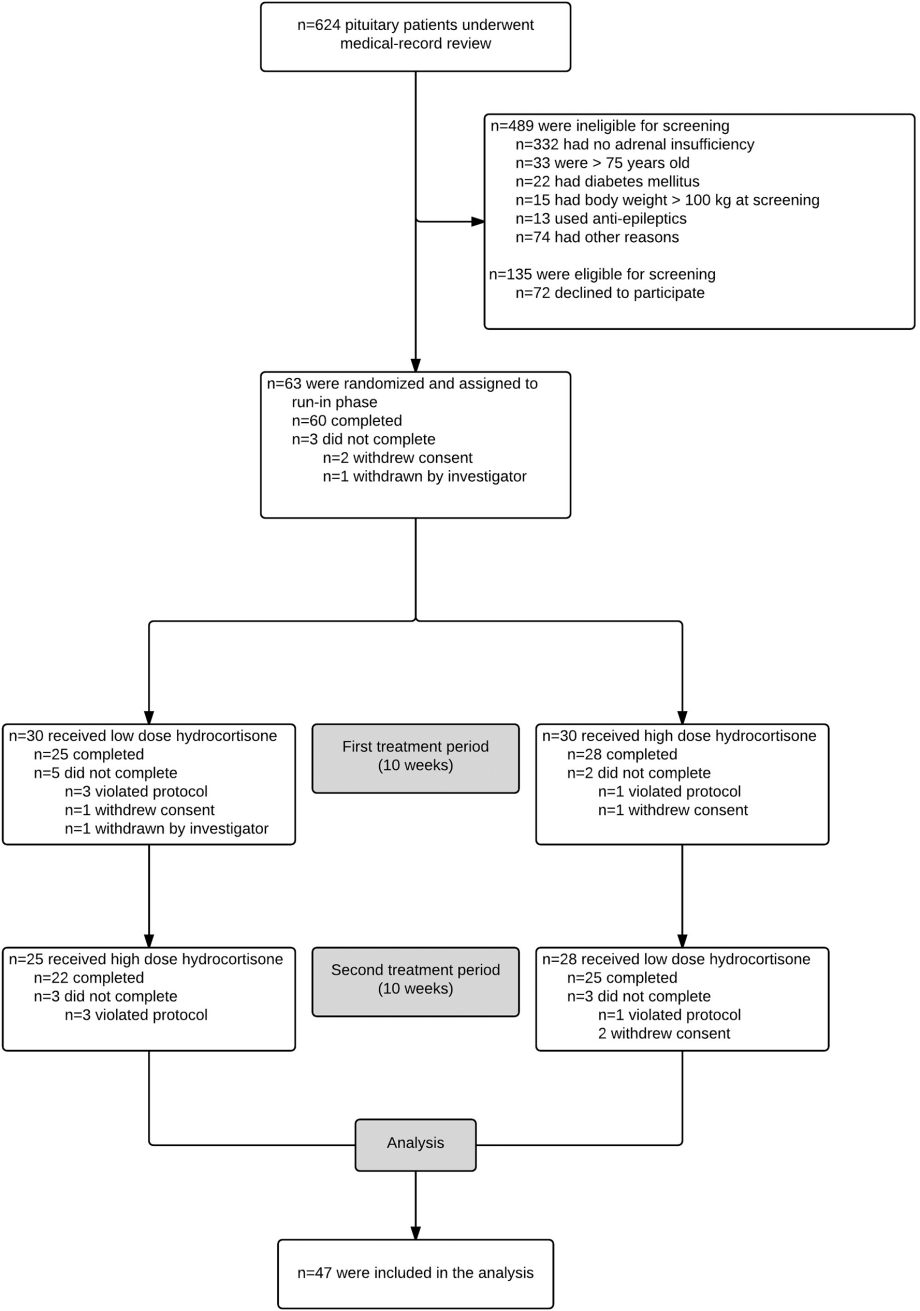
Patient flow diagram.

This study is part of a randomized double blind cross-over study [13]. Patients with SAI were recruited from the endocrine outpatient clinic at the University Medical Center Groningen (UMCG) between May 2012 and June 2013. Details about eligibility, recruitment and follow-up are shown in Fig 1. All patients were diagnosed with SAI according to internationally accepted criteria. Other inclusion criteria were age between 18 and 75 years, body weight between 50 and 100 kg, at least one year between treatment for pituitary disorder (surgery and/or radiotherapy) and study entry and stable replacement of other pituitary hormonal deficiencies for at least 6 months prior to study entry and during the study periods (thyroid hormone deficiency, growth hormone deficiency, testosterone/estradiol deficiency, diabetes insipidus) [13]. Main exclusion criteria were major cognitive impairment, drug abuse or dependence, current psychiatric disorders, malignancy, shift work, previous Cushing’s disease, hospital admission, medically treated diabetes mellitus potentially leading to hypoglyceamia (insulin, sulfonylurea derivatives), a history of frequent episodes of hypocortisolism and anti-epileptic drugs [13]. Forty-seven patients completed all testing days, QST data was available for 46 patients. Patients were allowed to use paracetamol or NSAIDs during the study. In total six patients reported the incidental use of these drugs but not on the test day and one participant reported the daily use of paracetamol/codeine. One patient used transdermal fentanyl and was excluded from the present analysis, resulting in a total patient group of 45 patients for the present analysis. The study protocol was approved by the Ethical Review Board of the University Medical Center Groningen, The Netherlands. Patients gave written informed consent before entering the study. This study is registered with Clinicaltrials.gov, number NCT01546922.

### Intervention and protocol

Patients were randomly assigned to either group 1 or group 2. Group 1 received first a low dose of HC (total daily dose of 0.2–0.3 mg/kg body weight) for 10 weeks, followed by a high dose of HC (total daily dose of 0.4–0.6 mg/kg body weight) for another 10 weeks. Group 2 received the two doses in reversed order. Randomization to one of the two treatment groups was performed by Tiofarma Inc, The Netherlands, with a block size of 4. The total daily dose was divided into three doses, administered before breakfast, before lunch and before dinner, with the highest dose given in the morning. After each treatment period patients returned to the hospital for evaluation. On testing days, patients were instructed to take their morning dose at 0700 hour. At 0800 hour the first blood samples were drawn in a fasting state in sitting position after a short period of rest, followed by physical, neuropsychological and sensory functional examination. Approximately five hours after the morning intake of HC a second blood sample was drawn.

### Laboratory measurements

Total cortisol concentrations in plasma was measured by isotope dilution liquid chromatography tandem mass spectrometry (LC-MS/MS) and was performed essentially as described by Hawley et al., using cortisol-13C_3_ as internal standard [21]. Intra- and inter-assay CVs were < 2.6% and <5.3%, respectively.

### Quantitative sensory testing

The QST battery utilized in the present study consisted of four tests and was applied on the hand (dorsum) according to the standardized protocol of Rolke et al. [16]. The QST battery was performed as part of an extensive neuropsychologic assessment battery. Testing and scoring was performed by trained personnel, under supervision of two neuropsychologists (PB en JK) to ensure a high standard and a minimal variation in test procedures. Not every QST parameter was thought to be relevant for the present patient group, for example dynamic mechanical allodynia and wind up ratio are used to assess central components of sensory function in neuropathic pain which was not expected to be present in the present patient population. In addition, in order not to exceed the time constraint of four hours of testing, only the following subset of mechanical tests of the QST battery were used:

The mechanical detection threshold (MDT) was measured with a standardized set of modified von Frey hairs (Optihair_2_-Set, Marstock Nervtest, Germany). Using the ‘method of limits’, five ascending and five descending series of stimulus intensities were applied, resulting in ten thresholds. The final threshold is the geometric mean of these ten thresholds. The mechanical pain threshold (MPT) was measured using a set of seven pinprick devices (flat contact area of 0.2 mm in diameter) with fixed stimulus intensities that exerted forces of 8, 16, 32, 64, 128, 256 and 512 mN. Using the ‘method of limits’, patients were instructed to indicate whether or not they perceived a sharp sensation. The final threshold was determined in equivalence to the procedure described for MDT, i.e. the geometric mean of ten thresholds. Mechanical pain sensitivity (MPS) was assessed using the same set of seven pinprick stimuli to obtain a stimulus-response function for pinprick-evoked pain. Five runs were performed and in each run the pinpricks were applied in balanced and pseudo-randomized order. Patients were asked to give a pain rating for each stimulus on a numerical rating scale ranging from ‘0’ (“no pain”) to ‘100’ (“most intense pain imaginable”). MPS was calculated as the geometric mean of all numerical ratings for pinprick stimuli. The pressure pain threshold (PPT) was determined over muscle with a pressure gauge device (FDN200, Wagner Instruments, USA) with three series of ascending stimulus intensities, each applied as a slowly increasing ramp of 50 kPa/s (0.5 kg/cm2 s). The final threshold was the geometric mean of three stimulations.

### Statistics

The present study is a post-hoc analysis of our primary study on cognitive functioning after the two different doses of hydrocortisone [13]. The power analysis performed for the study was based on this primary endpoint of the study. Based on a Wilcoxon Signed Rank Test, a study arm with each 25 patients (50 patients in total) would be able to detect a Cohen’s *d* effect size of 0.4 with a two-sided α = 0.05 and β = 0.80. In order to allow a dropout rate of approximately 20%, a total of 60 patients had to be included, as described previously [13]. All patients for which quantitative sensory testing data was available were included in the present analysis. QST data were transformed into z-scores as described by Rolke et al. [16], using age- and sex-specific reference values obtained in healthy volunteers [22]. A z-score below -1.96 or above 1.96 was considered a sensory abnormality. Normality of data was analyzed using Q-Q plots. Since the data were not normally distributed, differences in z-scores on the QST parameters between the two doses of HC were assessed using the non-parametric Wilcoxon Signed Rank Test for paired observations. In addition, Cohen’s *d* effect sizes were calculated to give a measure of the magnitude of the difference. An effect size of *d* = 0.2 is considered to be a small effect, *d* = 0.5 a moderate effect, and *d* = 0.8 a large effect [23]. Furthermore, the number of patients showing a sensory abnormality on each dose of HC were compared using the McNemar test. The two-tailed significance level of <0.05 was used. All statistical analyses were performed using Statistical Package for the Social Sciences (SPSS, Inc., Armonk, NY, USA), version 22.

## Results

### Patients

Reporting of the study conforms to the CONSORT 2010 statement [24]. Baseline characteristics are given in Table 1. A detailed description of the patient group and reasons for withdrawal can be found elsewhere [13].

**Table 1:** Clinical characteristics at baseline of pituitary patients treated for adrenal insufficiency (N = 45).

Sex (men/women), n	27/18
Age (y), median [IQR]	55 [43; 60]
Age at diagnosis (y), median [IQR]	31 [20; 45]
Body weight (kg), median [IQR]	81.5 [71.9; 93.1]
Hydrocortisone treatment prior to randomization	
Total daily dose (mg/day), median [IQR]	25 [20; 30]
Dose/kg body weight (mg/kg), median [IQR]	0.31 [0.25; 0.35]
Number of daily dosages (1/2/3), n	2/33/10
Duration of glucocorticoid treatment (y), median [IQR]	12 [6; 24]
No. of hormonal replacements (1/2/3/4/5), n	3/9/20/11/2
Thyroid hormone deficiency (% of patients)	91
Growth hormone deficiency (% of patients)	67
Growth hormone deficiency (% of patients receiving substitution)	67
Sex hormone deficiency (% of patients)	56
Men: testosterone (% of patients receiving substitution)	78
Premenopausal women, n = 8: estrogens (% of patients receiving substitution)	50
Postmenopausal women, n = 10: estrogens	NA
Desmopressin (% of patients)	18

Abbreviations: IQR: Interquartile range, NA: Not applicable.

### Cortisol levels

One hour after the administration of the morning dose, the higher dose of HC resulted in significantly increased plasma cortisol levels compared to the lower dose of HC (median [IQR], 741 [669; 870] nmol/L and 499 [383; 605] nmol/L for the higher and the lower dose respectively, P < 0.001). A similar difference was found five hours after administration of the morning dose (235 [170; 314] nmol/L and 112 [75; 199] nmol/L for the higher and the lower dose respectively, P < 0.001).

### Quantitative sensory testing

**Fig 2:**
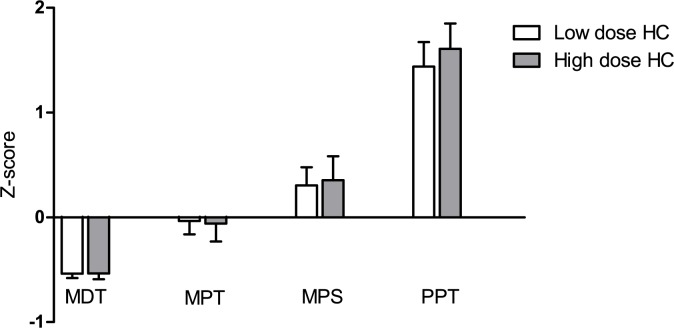
Z-scores for the mechanical detection threshold, mechanical pain threshold, mechanical pain sensitivity and pressure pain threshold on the lower dose and the higher dose (N = 45). Abbreviations: HC: hydrocortisone; MDT: mechanical detection threshold (P = 0.454 for the lower dose versus the higher dose); MPT: mechanical pain threshold (P = 0.800 for the lower dose versus the higher dose); MPS: mechanical pain sensitivity (P = 0.926 for the lower dose versus the higher dose); PPT: pressure pain threshold (P = 0.495 for the lower dose versus the higher dose). Data are mean ± SEM.

There were no differences in z-scores on the MDT, MPT, MPS and the PPT between the two doses of HC (Fig 2). Effect sizes ranged between 0.01 and 0.11. Furthermore, the number of patients showing at least one sensory abnormality did not differ between the lower and the higher dose of HC (29% (95% confidence interval 16–42%) of patients on the lower dose versus 27% (14–40%) of patients on the higher dose).

## Discussion

In this randomized double blind cross-over study, the effect of dose of HC on mechanical pain perception were studied. The administration of two different doses of HC for a period of 10 weeks did not result in a difference in mechanical QST parameters. This suggests that dose of HC does not alter mechanical pain perception in patients with SAI.

Other studies using drug administration to investigate the relationship between cortisol and pain sensitivity have revealed conflicting results. Some studies reported an association between cortisol administration and reduced pain [25], whereas others did not find altered pain sensitivity and pain thresholds after administration of exogenous cortisol [11,26]. Several studies used drugs to suppress the endogenous cortisol production to establish a state of hypocortisolism. Dexamethasone administration led to lowered pain thresholds [27]. However, next to decreased cortisol levels, dexamethasone administration leads to decreased ACTH or CRH, which could also result in reduced pain thresholds. Furthermore, reduced mechanical pain detection thresholds and enhanced temporal summation of pressure pain were reported in a hypocortisolemic state after oral administration of metyrapone [12]. However, the decrease in cortisol was accompanied by an increase in adrenocorticotropic hormone (ACTH) and possibly by other opioid proopiomelanocortin-derived peptides (including β-endorphin) [28]. Hypocortisolism and concomitant endorphin hyper-secretion can lead to hyperalgesia due to both insufficient anti-inflammatory glucocorticoid action and chronic opioid-induced activation of pro-inflammatory neuro-immune responses [29], therefore these peptides and mechanisms also might have played a role in the relationship between hypocortisolism and increased sensitivity of pain as found by Kuehl et al., (2010). Furthermore, Kuehl et al., (2010) assessed somatosensory function in healthy individuals, making a direct comparison with the results of the patient population of the present study difficult.

Despite the previously observed difference in subjective pain reports as assessed by daily diaries comparing both doses of HC [14], sensory changes supportive for HC induced alteration of mechanical function could not be established in this study. Therefore, previously found altered subjective pain due to a low dose of HC in SAI patients is not caused by different somatosensory functioning in response to mechanical stimuli. A methodological difference might underlie the discrepancy in findings. Previously, a poor relationship between clinical pain and pain thresholds measured with QST was reported [30]. A possible explanation for this poor association is that both methods measure different constructs of pain and may not be directly related. QST is a psychometric measure of evoked pain. However, mechanically induced pain is not necessarily the same as clinical pain in which patients refer to ongoing pain without an evocative stimulus. For example, in people with osteoarthritis of the knee it was shown that during spontaneous pain, i.e. pain experienced without stimulation, other brain areas were activated than brain areas that were active during evoked pain [31]. This implies that by measuring evoked pain, such as with mechanical stimuli, a differently constructed pain is experienced by patients without such a stimulation. Furthermore, even though the administration of the two different doses of HC in the present study resulted in significant and clinically relevant differences in plasma cortisol levels, the overall cortisol concentrations remained within the physiological range. In contrast, the state of hypocortisolism aimed for in the study by Kuehl et al. [12] can be considered subphysiological. This conceptual difference in study design could be an explanation for the lack of differences in QST parameters in our patient group.

The complete QST battery is a comprehensive test battery for somatosensory functioning across the full spectrum of nerve fibers: Aβ-fiber function, Aδ-fiber function and C-fiber function [16,17]. Aβ-fiber function is represented by the MDT and detection threshold of vibration; Aδ-fiber function is represented by the cold detection threshold and MPT. C-fiber function is represented by the warm detection threshold and heat pain threshold. The relative contribution of C- and Aδ-fiber nociceptors to cold and PPT is less clear. Due to time constraint the present study focused on the MDT, MPT, MPS, and PPT. As a consequence, C-fiber function, among others involved in heat sensation, was less extensively studied. However, in a study evaluating the influence of the order of thermal and mechanical tests on QST results in healthy volunteers, MPS was found to be increased significantly when thermal stimuli preceded mechanical testing [32]. Therefore, by excluding the thermal parameters, our mechanical results might result in a better representation of the thresholds as they are not influenced by sensitization due to preceding thermal stimuli.

A strength of the present study is the unique patient population enabling a comparison of the direct relation between cortisol levels and mechanical pain perception by excluding potential interference of the negative feedback mechanism of the HPA axis. A limitation is the small number of patients, which should be considered when interpreting the results of this study. However, given the negligible effect sizes, we do not expect to find different results in a larger sample. Even though we’ve performed multiple tests, we did not adjust for the inflated type I error. However, the null findings of the results even without adjusting for multiple testing underscores that there is no effect. Furthermore, 10 weeks of treatment might be too short to find dose-dependent effects of HC on mechanical pain perception. However, the study by Kuehl et al. (2010) showed significant differences in sensory perception as soon as six hours after induced hypocortisolism, so a lack of differences due to a too short treatment period is in our opinion unlikely. One can speculate that plasma levels are of importance, but the main effects of hydrocortisone are genomic and temporal relationships with several endpoints including somatosensory functioning may therefore differ from plasma concentrations.

To the best of our knowledge, this is the first study examining the effect of two different doses of HC on mechanical parameters in patients with SAI. Our results indicate that the dose of HC has no impact on mechanical pain perception in patients with SAI, even though a difference in subjectively experienced pain symptoms was reported previously by the same patient group [14].

## Supporting information

S1 Text (DOCX)

S2 Text (PDF)
