# Supplementary material for: Somatosensory function in patients with secondary adrenal insufficiency treated with two different doses of hydrocortisone—Results from a randomized controlled trial
Source: PLoS One. 2017 Jul 7;12(7):e0180326. doi: 10.1371/journal.pone.0180326 (PMC5501533; doi:10.1371/journal.pone.0180326)
Supplement: S2 Text — (PDF) [file pone.0180326.s002.pdf]

A randomized double blind cross-over study of the effects of low dose and high dose hydrocortisone replacement therapy on cognition, quality of life, metabolic profile and somatosensation in patients with secondary adrenal insufficiency

Version 3, August 2012

PROTOCOL TITLE

A randomized double blind cross-over study of the effects of low dose and high dose hydrocortisone replacement therapy on cognition, quality of life, metabolic profile and somatosensation in patients with secondary adrenal insufficiency.

Protocol ID	HydroCog
Short title	Hydrocortisone replacement in patients with secondary adrenal insufficiency.
Work title	SUbstitution with high or low Physiological REplaceMEnt doses of hydroCORTisone: The SUPREME CORT study
Version	3
Date	August, 2012
Coordinating investigator/ Project leader	Dr. A.P. van Beek<br>University Medical Center Groningen (UMCG)<br>De Brug, 4.069<br>Hanzeplein 1,<br>9700 RB Groningen<br>a.p.van.beek@umcg.nl<br>Tel. 050-3610388
Principal investigator(s):<br>(in Dutch: hoofdonderzoeker/ Uitvoerder)	Dr. A.P. van Beek<br>University Medical Center Groningen (UMCG)<br>De Brug, 4.069<br>Hanzeplein 1,<br>9700 RB Groningen<br>a.p.van.beek@umcg.nl<br>Tel. 050-3610388<br><br>P. Brummelman, MSc.<br>University Medical Center Groningen (UMCG)<br>De Brug, 4.065<br>Hanzeplein 1,<br>9700 RB Groningen<br>p.brummelman@umcg.nl<br>Tel. 050-3612978
Sponsor(in Dutch: verrichter/ opdrachtgever)	University Medical Center Groningen<br>Hanzeplein 1<br>Postbus 30.001<br>9700 RB Groningen
Independent physician	Dr. A.N.A. van der Horst<br>University Medical Center Groningen (UMCG)<br>De Brug, 4.063<br>Hanzeplein 1,<br>9700 RB Groningen<br>a.n.a.schrivers@umcg.nl<br>Tel. 050-3610542
Laboratory sites	Not applicable
Pharmacy	TioFarma BV<br>Benjamin Franklinstraat 10<br>3261 LW Oud Beijerland<br>Tel.nr.: 0186-614778

	Fax.: 0118-621832<br>E-mail: info@tiofarma.nl<br>Website: http://www.tiofarma.nl

PROTOCOL SIGNATURE SHEET

Name	Signature	Date
**Sponsor or legal representative:**<br>University Medical Center Groningen<br>Hanzeplein 1<br>Postbus 30.001<br>9700 RB Groningen<br><br>For non-commercial research,<br>Head of department:	Dr. A.P. van Beek<br>Internist endocrinologist<br><br>Prof. dr. B.H.R. Wolffenbuttel<br>Internist endocrinologist	
Coordinating investigator:	Dr. A.P. van Beek<br>Internist endocrinologist	

TABLE OF CONTENTS

1. INTRODUCTION AND RATIONALE 9
2. OBJECTIVES....11
3. STUDY DESIGN 11
4. STUDY POPULATION....11
4.1 Population (base)....11
4.2 Inclusion criteria....11
4.3 Exclusion criteria....12
4.4 Sample size calculation....12
5. TREATMENT OF SUBJECTS....13
5.1 Investigational product/treatment 13
5.2 Use of co-intervention 13
5.3 Escape medication....13
6. INVESTIGATIONAL MEDICINAL PRODUCT....14
6.1 Name and description of investigational medicinal product....14
6.2 Summary of findings from non-clinical studies....14
6.3 Summary of findings from clinical studies....14
6.4 Summary of known and potential risks and benefits....14
6.5 Description and justification of route of administration and dosage....14
6.6 Dosages, dosage modifications and method of administration 14
6.7 Preparation and labelling of Investigational Medicinal Product 15
6.8 Drug accountability 15
7. METHODS....16
7.1 Study parameters/endpoints 16
7.1.1 Main study parameter/endpoint....16
7.1.2 Secondary study parameters/endpoints 16
7.1.3 Other study parameters....16
7.2 Randomisation, blinding and treatment allocation 16
7.3 Study procedures....17
7.4 Withdrawal of individual subjects 19
7.5 Replacement of individual subjects after withdrawal 19
7.6 Follow-up of subjects withdrawn from treatment....19
7.7 Premature termination of the study 19
8 SAFETY REPORTING 20
8.1 Section 10 WMO event 20
8.2 Adverse and serious adverse events 20
8.2.1 Suspected unexpected serious adverse reactions (SUSAR) 20
8.2.2 Annual safety report.... 21
8.3 Follow-up of adverse events....21
8.4 Data Safety Monitoring Board (DSMB).... 21
9. STATISTICAL ANALYSIS 21
9.1 Descriptive statistics 21
9.2 Treatment analyses 21
10. ETHICAL CONSIDERATIONS....22
10.1 Regulation statement....22
10.2 Recruitment and consent 22
10.3 Objection by minors or incapacitated subjects (if applicable)....22
10.4 Benefits and risks assessment, group relatedness....22
10.5 Compensation for injury 22
10.6 Incentives (if applicable)....22
11. ADMINISTRATIVE ASPECTS AND PUBLICATION 23
11.1 Handling and storage of data and documents....23
11.2 Amendments....23
11.3 Annual progress report....23

11.4 End of study report..23
11.5 Public disclosure and publication policy 23
12. REFERENCES………………………………………………………………………… 24
13. Appendix…………………………………………………………………………….27
Appendix 1 Cognitive Test Battery……………………………………………………..27
Appendix 2 The daily mood and symptom report…………………………………….28
Appendix 3 Instructions for the QoL Questionnaires…………………………………34
Daily activities…………………………………………………………………35
Rand-36………………………………………………………………………….36
Welfare (HADS)……………………………………………………………….40
Fatigue (MVI-20)……………………………...………………………………42
Cognition (CFQ)……………………………………………………………….44
Personal Information………………………………………………………….47
Appendix 4 Somatosensation – Quantitative sensory testing……………………………….49

LIST OF ABBREVIATIONS AND RELEVANT DEFINITIONS

15 WT	15 words test
ABR	ABR form, General Assessment and Registration form, is the application form that is required for submission to the accredited Ethics Committee (In Dutch, ABR = Algemene Beoordeling en Registratie)
AE	Adverse Event
AR	Adverse Reaction
BB	Before breakfast
BL	Before lunch
BD	Before dinner
CA	Cortisonacetate
CCMO	Central Committee on Research Involving Human Subjects; in Dutch: Centrale Commissie Mensgebonden Onderzoek
CFQ	Cognitive Failure Questionnaire
CV	Curriculum Vitae
DSMB	Data Safety Monitoring Board
EU	European Union
EudraCT	European drug regulatory affairs Clinical Trials
GC	Glucocorticoïd
GCs	Glucocorticoïds
GCP	Good Clinical Practice
HADS	Hospital Anxiety and Depression Scale
HC	Hydrocortisone
HPA-axis	Hypothalamus-Pituitary-Adrenal axis
IB	Investigator’s Brochure
IC	Informed Consent
IMPD	Investigational Medicinal Product Dossier
M1	Measurement 1
M2	Measurement 2
M3	Measurement 3
METC	Medical research ethics committee (MREC); in Dutch: medisch ethische toetsing commissie (METC)
MFI	Multidimensional Fatigue Inventory
mLLT	modified Location Learning Test
MVI	Multidimensionale Vermoeidheidsindex
PFC	Prefrontal cortex

QST	Quantitative Sensory Testing
QofL	Quality of Life
RBMT	Rivermead behavioral memory test
RMET	Reading the mind in the eyes test
SD	Standard deviation
SAE	Serious Adverse Event
SPC	Summary of Product Characteristics (in Dutch: officiële productinfomatie IB1-tekst)
Sponsor	The sponsor is the party that commissions the organisation or performance of the research, for example a pharmaceutical company, academic hospital, scientific organisation or investigator. A party that provides funding for a study but does not commission it is not regarded as the sponsor, but referred to as a subsidising party.
SUSAR	Suspected Unexpected Serious Adverse Reaction
Wbp	Personal Data Protection Act (in Dutch: Wet Bescherming Persoonsgevens)
WMO	Medical Research involving Human Subject Acts (Wet Medisch Wetenschappelijk Onderzoek met mensen)

SUMMARY

Rationale:
A wide variety in hydrocortisone (HC) substitution dose-regimens are considered physiological for patients with adrenal insufficiency. However, it is likely that cognition is negatively influenced by higher cortisol exposure to the brain. No studies have been performed to assess the effects of treatment regimens with a low physiological HC substitution dose on cognition in comparison to a high physiological HC substitution dose. These treatment regimens should take body weight and multiple dosing into account. In addition, substitution doses should be monitored by clinical evaluation and biochemical analysis for adverse effects associated with over- or under-replacement.

Objective:
The aim of this study is to investigate whether a physiologically low HC dose is better for cognition as compared to a high HC dose. In addition quality of life, metabolic profile and somatosensation will be described in relation to HC dose.

Study design: Randomized, double blind cross-over design.

Study population: Sixty-six patients diagnosed with secondary adrenal insufficiency on conventional replacement therapy. Participants will be 18-75 years old and on stable hormonal replacement therapy for at least six months.

Intervention:
Patients will be randomized in two groups matched for sex and body weight to receive either a low dose HC (0.2-0.3 mg/kg body weight) for 10 weeks followed by 10 weeks of high dose HC (0.4-0.6 mg/kg body weight) or high dose HC followed by a low dose of HC.

Main study parameters/endpoints:
The primary endpoint is cognitive performance. The secondary endpoints are quality of life including somatic complaints during treatment, metabolic profile and somatosensation.

Nature and extent of the burden and risks associated with participation, benefit and group relatedness:
Burden: At baseline and after completion of both treatment arms patients will undergo neuropsychological evaluation. During these 3 visits (duration ± 4 hours for each visit) they will also fill in quality of life questionnaires. Blood samples will be drawn before and after the test battery. During both treatment periods patients will keep a diary regarding common somatic complaints and mood.
Risks: All HC dosing schemes can be considered safe and are published in literature. Additional hydrocortisone dose escalation is allowed to prevend hypocortisolism. The risk of severe hypocortisolism on study dose-regimens of HC is small and estimated to be similar to conventional treatment at the outpatient clinic.
Benefit: participant's potential preference to either dosing scheme in addition to insight in risks and benefit of high or low dose HC treatment.

1. INTRODUCTION AND RATIONALE

Patients with adrenal insufficiency are treated with glucocorticoids (GCs) to compensate for the loss of endogenous cortisol production. Usually this is done by oral administration of hydrocortisone (HC) or cortisonacetate (CA). The aim is to mimic the endogenous cortisol rhythm, with peak values in the early morning before waking and a nadir at bedtime. In many countries HC is generally considered to be the first choice for a glucocorticoid (GC) regimen, because CA must undergo hepatic conversion to cortisol to become active.

HC substitution dose and regimens has varied over the previous decades. Initial doses of 30 mg/day were based on previously estimated values of cortisol production rates of 12-15 mg/m^2/day, resulting in over-replacement (1). Currently, endogenous cortisol production rate in adults with intact adrenal reserve is estimated to approximate 6–10 mg/m^2 /day (2;3). As a result a wide variety of replacement doses is recommended. However, evidence comes from observational studies or unsystematic clinical experience and is graded as a very weak recommendation (grade 2C) because of absence of randomized controlled trials (4). Therefore, current practice varies widely, as evidenced by our own outpatient clinic where approximately half of the patients are on low physiological GC substitution while the other half is treated with a high physiological GC dose (5).

It is generally suggested to use the lowest GC dose that relieves symptoms of GC deficiency and avoids signs and symptoms of GC excess. Some advocate the use of normative day-curve cortisol values for the assessment of the adequacy of hydrocortisone therapy (6) but others suggest that clinical assessment alone works equally well (7). If the replacement dose is too low symptoms of apparent glucocorticoid deficiency are present (f.e. hypoglycemia, muscle weakness). In contrast, if the dose is too high excessive weight gain, hypertension and dyslipidemia may be present.

The effects of hydrocortisone substitution are likely to depend on body weight, as this was found to be the most important variable determining hydrocortisone clearance (6). Experts recommend a weight-adjusted hydrocortisone dose of 0.12 mg/kg body weight for the morning dose (1). Thrice-daily weight adjusted administration mimics the day-curve of cortisol seen in healthy volunteers and is necessary because of the short half-life (6).

GC substitution therapy, although referred to as physiological, has its imperfections. Current GC dose-regimens inevitably result in over- or under-replacement during certain periods of the day. This may result in poor quality of life (1). In addition, some non-specific symptoms, such as fatigue and headache in the early morning, are frequent in this group of patients (8). Filipsson and colleagues found that GC substitution, especially in higher physiological doses (> 20 mg/day) was associated with an unfavorable metabolic profile when compared with patients with normal adrenal function (9).

Besides physical side effects, mental side effects are reported in patients treated with pharmacological doses of GC, or in healthy individuals. With regard to cognition, especially deficits in memory and executive functioning are reported at higher cortisol doses as illustrated in the next two paragraphs. However, evidence is mainly derived from healthy volunteers with normal adrenal function (10-20).

There is ample evidence that GCs are necessary for learning and memory in humans (10-13;15;16;18;19;21-23). At least in healthy humans, elevation of basal cortisol levels is associated with reduced hippocampal volume and impairments on learning and memory tasks which depend upon the integrity of the hippocampus (22). Memory can be divided in emotional and neutral memory, i.e. respectively memory for events with or without an emotional charge (that can be the valence of the stimulus or the context of an event). Glucocorticoïds have been found to affect emotional memory, likely through actions in the amygdala (22;24). Indeed, Maheu and colleagues demonstrated that the valence of the to-be-remembered material, modulates the effects of stress on human declarative memory (i.e. knowledge that we have conscious access to, including personal and world knowledge) (25). Furthermore, emotionally arousing and stressful experiences influence declarative memory likely through their interaction with GC receptors located in the frontal lobes, amygdala and hippocampus (26-29).

Executive functions also appeared to be sensitive to the variations of GCs (14;17;20;30). The prefrontal cortex, which is strongly associated with executive functioning**,** contains a high concentration of glucocorticoïd receptors (31) and is an important site for regulation of the Hypothalamus-Pituitary-Adrenal-axis (32). Thus it is reasonable to expect that GCs would have an impact on executive functions, such as working memory and that there exist a quadratic function between GCs and executive function (14). Working memory was found to be more sensitive to the acute (i.e. given by infusion) effects of GCs than declarative memory (14). Working memory is important for acquisition and consolidation of information and therefore for all kind of memories. In addition, cortisol may lead to less salient encoding of meaningful stimuli and may impair selective attention, thereby reducing an individual's ability to discriminate relevant and important information from irrelevant and unimportant information (23). However, later studies suggest that cortisol does not affect attention (10;19). Because attention is of great importance for almost all cognitive functions, it is important to know the effects of GCs on attention. Furthermore we will perform some quantitative sensory testing (QST), including thermal as well as mechanical testing procedures (33). We expect increased basal mechanical pain sensitivity but decreased perceptual wind-up in patients in the low dose group compared to the high dose group, based on a study by Kuehl and colleagues (34).

In conclusion, a wide variety in HC substitution dose-regimens is considered physiological for patients with adrenal insufficiency. However, it is likely that cognition is negatively influenced by higher cortisol exposure to the brain. No studies have been performed to assess the effects of treatment regimens with a low physiological HC substitution dose on cognition in comparison to a high physiological dose. These treatment regimens should take body weight and multiple dosing into account. In addition, substitution doses should be monitored by clinical evaluation and biochemical analysis for adverse effects associated with over- or under-replacement.

Hypotheses:

We hypothesize that a low physiological HC dose results in better cognitive performance and improved metabolic risk profile, but in decreased quality of life with excess common somatic complaints and increased values of somatosensation in patients with secondary adrenal insufficiency when compared to a high physiological HC dose.

Study Aim:

In this double blind cross-over study we will investigate the effects of a low physiological HC substitution on cognition, quality of life, metabolic profile and somatosensation, in patients with secondary adrenal insufficiency and compare them to a high physiological HC substitution dose.

2. OBJECTIVES

Primary objective: to study the effects of a low versus high physiological HC substitution on cognition.

Secondary objectives: to study the effects of a low versus high physiological HC substitution on:
- Quality of life (QofL),
  -common somatic complaints in relation to mood
- Metabolic profile
- Somatosensation

3. STUDY DESIGN

This is a randomized double blind cross-over trial of the effects of low dose and high dose hydrocortisone replacement therapy.

The protocol can be summarized as below:

	**Run-in phase** *(4weeks)*	**M1**	*10 weeks*	**M2**	*10 weeks*	**M3**
**Group A**	Prestudy dose→ bioequivalent HC dose*		High dose		Low dose	
**Group B**	Prestudy dose→ bioequivalent HC dose*		Low dose		High dose	

M1: measurement 1, baseline evaluation
M2: measurement 2, evaluation of first treatment dose
M3: measurement 3, evaluation of second treatment dose

* In the run-in-phase all patients on CA participating in this study will be converted to treatment with HC in a bioequivalent dose to avoid effects of switching to a different type of glucocorticoid on the first treatment dose.
The bioequivalence of CA = 0.8 when compared to HC.

Patients will be treated for 10 weeks after which a switch in study dose will take place.

4 STUDY POPULATION

4.1 Population (base)

Approximately, 400 patients with secondary adrenal insufficiency without prior evidence of hormonal overproduction (e.g. acromegaly, Cushing's disease and prolactinoma) have a regular follow-up at the Endocrine outpatient clinic at the UMCG. Because of this number we think that it is feasible to collect 66 out of 400 patients who will meet the inclusion criteria. Patients included in this study will be 18-70 years old, all on stable GC replacement for at least six months.

4.2 Inclusion criteria

Patients with secondary adrenal insufficiency
Age ≥ 18 – 75 years
≥ One year after tumor treatment with surgery and/or radiotherapy
On stable concomitant medications for at least six months prior to entry of study
Body weight 50-100 kg

4.3 Exclusion criteria

Inability of legal consent
Documented cognitive impairment
Drug abuse/dependence
History of / current psychiatric disorders
Use of anti-epileptics (e.g. carbamezapine)
Cushing disease
Type 1 or Type 2 diabetes
Current treatment for second malignancy
Have a significant medical condition (e.g. hepatic, respiratory, or cardiovascular) which, in the opinion of the investigator, may interfere with the interpretation of results and safety or efficacy evaluations.
A history of frequent hypocortisolism
Hospitalization during study
Work in shifts

The in- and exclusion criteria of potential candidates will be checked by the physician responsible for the endocrine outpatient care and by information available in the medical records. In the Informed Consent form, patients can give permission if they agree with the fact that other people (as stated in the general brochure) can see their medical data.

4.4 Sample size and calculation

No relevant data can be inferred from literature to estimate a reasonable treatment effect of HC dose in secondary adrenal insufficiency on cognition.
Because of the absence of relevant data from literature, we chose to perform a power analyses.

A study with 2 arms with each 25 patients (total number of patients: 50) is able to detect an effect size of 0.4 (two sided $\alpha = 0.05$ and $\beta = 0.80$) in test results even when between test correlations are poor (0.50).

An effect size of 0.4 was chosen because it is considered a relevant change in a small to medium size effect.

To allow for a drop-out rate of ±25% a total number of 66 patients is needed.

5. TREATMENT OF SUBJECTS

5.1 Investigational product/treatment

Patients with secondary adrenal insufficiency will be treated with *oral hydrocortisone* tablets adjusted to their weight according to the following dose-regimens: 0.2-0.3 mg/kg body weight/day or 0.4-0.6 mg/kg body weight/day in three divided doses.

5.2 Use of co-intervention

Subjects are allowed to use co-medication, except for the drugs that are mentioned in the exclusion criteria.

5.3 Escape medication

Patients are allowed to double or triple their HC dose in cases of small medical operations (e.g. when visiting a dentist) or in cases of fever (body temperature > 38^0 C). Patients are already familiar with the escape medication rules which do not differ from regular practice at the Endocrine outpatient practice. Because the study aims to investigate two different dosing-schemes increasing the dose of HC is allowed for a maximum of 1 week (i.e. 10% of the study time) and not in the week preceding M2 or M3.

6. INVESTIGATIONAL MEDICINAL PRODUCT

6.1 Name and description of investigational medicinal product
Hydrocortisone

6.2 Summary of findings from non-clinical studies
Details can be found in the SPC page 6.

6.3 Summary of findings from clinical studies
Details can be found in the IMPD / SPC pages 1-6.

6.4 Summary of known and potential risks and benefits
Risks: All HC dosing schemes can be considered safe and are published in literature. Additional hydrocortisone escape medication to provide an imitation of physiological stress response is allowed. The risk of severe hypocortisolism on study dose-regimens of HC is small and estimated to be similar to conventional treatment at the outpatient clinic. Higher HC doses may slightly increase plasma glucose, triglycerides and blood pressure and may produce some weight gain. During a 10 week treatment period with the physiological treatment doses these changes are expected to be small.
Benefit: participant's potential preference to either dosing scheme in addition to insight in risks and benefit of high or low dose HC treatment.

6.5 Description and justification of route of administration and dosage
The route of administration is oral. The dosages are published dosage schemes and widely recommended by leading health organizations and expert groups. They can be inferred from endogenous cortisol production rates in healthy volunteers which are known to vary from 6-15 mg/m^2/dag. Thrice daily administration mimics the day-curve of cortisol seen in healthy volunteers. To mimic peak values in the early morning the highest substitution doses are before breakfast.

6.6 Dosages, dosage modifications and method of administration

Dosage

Group A - low HC condition

weight (kg)	BB	BL	BD	Total
50-74	7,5	5,0	2,5	15
75-84	10,0	5,0	2,5	17,5
85-100	10,0	7,5	2,5	20

Group B - high HC condition

weight (kg)	BB	BL	BD	Total
50-74	15,0	10,0	5,0	30
75-84	20,0	10,0	5,0	35
85-100	20,0	15,0	5,0	40

Dose in mg. BB: Before Breakfast. BL: Before Lunch. BD: Before Dinner.
Total: cumulative daily dose.

Dosage modification
Patients are allowed to double or triple their HC dose in cases of small medical operations (e.g. when visiting a dentist) or in cases of fever (body temperature > 38^0 C). Patients are already familiar with the escape medication rules which do not differ from regular practice at the Endocrine outpatient practice. Because the study aims to investigate two different dosing-schemes increasing the dose of HC is allowed for a maximum of 7 days (i.e. 10% of the study time) and not in the week preceding M2 or M3.

Method of administration
Oral tablets

6.7 Preparation and labelling of Investigational Medicinal Product
Tiofarma B.V. is responsible for labeling the hydrocortisone tablets.
The labels comply with annex 13 of the GMP; it contains all the requirements placed on the labels.

6.8 Drug accountability
Tiofarma B.V. is responsible for delivery of the study medication by courier to the pharmacy of UMCG. The pharmacy reviewes the medication and distrubutes them on the basis a study patient nummer provided by the prinicipal investigator.

Remaining tablets will be counted after each treatment period and compared to patient’s recording of HC tablet intake. Remaining drug tablets will be taken by the pharmacy and destroyed in the UMCG.

7. METHODS

7.1 Study parameters/endpoints

7.1.1. Main study parameter/endpoint
Cognitive performance.

7.1.2. Secondary study parameters/endpoints
Quality of Life (QoL),
- Common somatic complaints in relation to mood

Metabolic profile
Somatosensation

7.1.3 Other study parameters (if applicable)
Not applicable.

7.2 Randomisation, blinding and treatment allocation
This will be a randomized (group A or group B), double blind, cross-over study.

	**Run-in phase** (4weeks)	**M1**	*10 weeks*	**M2**	*10 weeks*	**M3**
**Group A**	Prestudy dose → bioequivalent HC dose		High dose		Low dose	
**Group B**	Prestudy dose → bioequivalent HC dose		Low dose		High dose	

Breaking the randomisation code is necessary in case of experienced serious discomfort in the patients' physical of mental functioning caused by the new hydrocortisone dose, and not resolving by temporary doubling or tripling of the HC dose. The pharmacy of the UMCG can break the randomisation code.
During a 10 week treatment period with the physiological treatment doses this is not expected to happen.

7.3 Study procedures

Patients from the Endocrine outpatient clinic are eligible for participation in this study. The study procedure is as outlined by the following schedule:

Protocol activity	Screening	Run-in Phase	M1	Study Treatment Period 1	M2	Study Treatment Period 2	M3
		28 days		70 days		70 days	
**Visit**	**0**		**1**		**2**		**3**
**Day**			**-1**		**70**		**140**
Informed consent	**-35**						
Demographics	x						
Concomitant Medication	x						
Medical History	x						
Physical Examination	x				x		x
Blood samples drawn			x		x		x
Randomize to treatment sequence			x				
Dispense treatment			x		x		
Study instructions and telephone numbers			x		x		
Telephone follow up (day)				**Day 35**		**Day 105**	
Cognitive test battery			x		x		x
Quality of Life questionnaires			x	**Day 69**		**Day 139**	
The Daily mood and symptom report (diaries during 70 days)				x		x	
Collecting beard hair in men					x		x
24 hr UFF measurement (day)				**Day 69**		**Day 139**	
Drug accountability					x		x
Assessment Adverse Events				x	x	x	x

Test procedure

Visit 1, 2 and 3 (M1, M2, and M3):

All three test visits will be performed according to the schedule below.

**Time**		**Place**	**Action**
start	end		
07.00h		Home	morning Hydrocortison 15 min before breakfast
08.00h	08.30h	Laboratory F18	blood samples drawn, hand in 24-h urine collection, collecting beard hair in men
08.30h	09.30h	Test Ward F17	Start Cognitive Test Battery (part 1)
09.30h	10.00h		Break (Tea or Coffee)
10.00h	11.00h	Test Ward F17	Start Cognitive Test Battery (part 2)<br>Physical examination + somatosensation
11.00h	11.15h	Laboratory F18	blood samples drawn
11.15h	11.45h	Test Ward F17	*study-phase specific activities*<br>M1: Dispense treatment, study instructions and telephone numbers. Provide diaries and urine collection material.<br>M2: Dispense treatment. Provide diaries and urine collection material. Assessment Adverse Events. Drug accountability.<br>M3: Assessment Adverse Events. Drug accountability.

Approximate duration of each visit will be 3 hours and 45 min.

Cognitive test Battery: see appendix 1.

The Daily mood and symptom report: see appendix 2.

Quality of Life questionnaires: see appendix 3.

Somatosensation: quantitative sensory testing: see appendix 4.

Physical examination
Height, body weight, waist- and hip circumference. Blood pressure was measured with an automated device (Dinamap XL Model 9300; Johnson & Johnson Medical, Tampa, FL).

Laboratory measurements

Blood

A. Before test battery: sodium, potassium, urea, creatinine, fT4, cortisol, IGF-1, FSH, LH, testosterone (♂ only), estradiol (♀ only), prolactin, glucose, HbA1c, cholesterol, triglycerides, HDL, cholesterol, LDL cholesterol, Hb, MCV leucocytes, CRP, ASAT, ALAT, LDH, AF, gammaGT, bilirubin.
   CTX + P1NP. Buffy coat for GC – Receptor genotype. Storage serum samples (EDTA and heparin).

B. After test battery: glucose, cortisol.

Total volume (A + B) 50 cc.

Urine
24-h urine collection for creatinin, free cortisol and steroid profile.

Hair
Beard hair will be collected with the help of a dry razor blade on the morning of M2 and M3 and stored dry at minus 80 degree until analyzed. Cortisol in hair is measured on the LCMS-MS (courtesy of Prof IP Kema).

7.4 Withdrawal of individual subjects
Subjects can leave the study at any time for any reason if they wish to do so without any consequences. The attending physician can decide to withdraw a subject from the study for urgent medical reasons.

7.5 Replacement of individual subjects after withdrawal
Not applicable.
In anticipation, we included ± 25 % more patients than we need to find significant differences as described in section 4.4. For this reason, patients will not be replaced when they decide to discontinue with the study.

7.6 Follow-up of subjects withdrawn from treatment
Withdrawal from the study has no consequences for further treatment. All patients, including withdrawn patients, will continue their regular visits to the department of Endocrinology of the UMCG.

7.7 Premature termination of the study
Premature ending of this study is not to be suspected. If there is a premature ending of the study it will not have any consequences for the treatment of patients.

8. SAFETY REPORTING

8.1 Section 10 WMO event

In accordance to section 10, subsection 1, of the WMO, the investigator will inform the subjects and the reviewing accredited METC if anything occurs, on the basis of which it appears that the disadvantages of participation may be significantly greater than was foreseen in the research proposal. The study will be suspended pending further review by the accredited METC, except insofar as suspension would jeopardize the subjects' health. The investigator will take care that all subjects are kept informed.

8.2 Adverse and serious adverse events

Adverse events are defined as any undesirable experience occurring to a subject during a clinical trial, whether or not considered related to the investigational drug. All adverse events reported spontaneously by the subject or observed by the investigator or his staff will be recorded.

A serious adverse event (SAE) is any untoward medical occurrence or effect that at any dose:

- results in death;
- is life threatening (at the time of the event);
- requires hospitalization or prolongation of existing inpatients' hospitalisation;
- results in persistent or significant disability or incapacity;
- is a congenital anomaly or birth defect;
- is a new event of the trial likely to affect the safety of the subjects, such as an unexpected outcome of an adverse reaction, lack of efficacy of an IMP used for the treatment of a life threatening disease, major safety finding from a newly completed animal study, etc.

All SAEs will be reported through the web portal *ToetsingOnline* to the accredited METC that approved the protocol, within 15 days after the sponsor has first knowledge of the serious adverse reactions.
SAEs that result in death or are life threatening should be reported expedited. The expedited reporting will occur not later than 7 days after the responsible investigator has first knowledge of the adverse reaction. This is for a preliminary report with another 8 days for completion of the report.

8.2.1 Suspected unexpected serious adverse reactions (SUSAR)

Adverse reactions are all untoward and unintended responses to an investigational product related to any dose administered.

Unexpected adverse reactions are adverse reactions, of which the nature, or severity, is not consistent with the applicable product information (e.g. Investigator's Brochure for an unapproved IMP or Summary of Product Characteristics (SPC) for an authorised medicinal product).

The sponsor will report expedited the following SUSARs through the web portal *ToetsingOnline* to the METC:

- SUSARs that have arisen in the clinical trial that was assessed by the METC;
- SUSARs that have arisen in other clinical trials of the same sponsor and with the same medicinal product, and that could have consequences for the safety of the subjects involved in the clinical trial that was assessed by the METC.

The remaining SUSARs are recorded in an overview list (line-listing) that will be submitted once every half year to the METC. This line-listing provides an overview of all SUSARs from the study medicine, accompanied by a brief report highlighting the main points of concern. The expedited reporting of SUSARs through the web portal ToetsingOnline is sufficient as notification to the competent authority.

The sponsor will report expedited all SUSARs to the competent authorities in other Member States, according to the requirements of the Member States.

The expedited reporting will occur not later than 15 days after the sponsor has first knowledge of the adverse reactions. For fatal or life threatening cases the term will be maximal 7 days for a preliminary report with another 8 days for completion of the report.

The pharmacy of the UMCG is able to break the code to find out whether the patient is in a low or high hydrocortisone condition.

8.2.2. Annual safety report

In addition to the expedited reporting of SUSARs, the sponsor will submit, once a year throughout the clinical trial, a safety report to the accredited METC, competent authority, Medicine Evaluation Board and competent authorities of the concerned Member States.
This safety report consists of:

- a list of all suspected (unexpected or expected) serious adverse reactions, along with an aggregated summary table of all reported serious adverse reactions, ordered by organ system, per study;
- a report concerning the safety of the subjects, consisting of a complete safety analysis and an evaluation of the balance between the efficacy and the harmfulness of the medicine under investigation.

8.3 Follow-up of adverse events

All adverse events will be followed until they have abated, or until a stable situation has been reached. Depending on the event, follow up may require additional tests or medical procedures as indicated, and/or referral to the general physician or a medical specialist.

8.4 Data Safety Monitoring Board (DSMB)

Not applicable

9 STATISTICAL ANALYSES

9.1 Descriptive statistics

Data on demographic and baseline characteristics will be summarized by mean (or median), standard deviation (or 25th or 75th percentiles), minimum and maximum for continuous variables and by proportions (percentages) for discrete variables.

9.2 Treatment analysis

Treatment effects will be assessed by analysis of differences between M2 and M3 using a *paired samples t-test* or a *non-parametric test* where appropriate. M1 serves as a reference within the cognitive functioning of patients at baseline (that is: on their conventional HC doses therapy).

In case of confounding variables – what we do not expect – we will decide to use a Linear Mixed Effect model.

10. ETHICAL CONSIDERATIONS

10.1 Regulation statement
This study will be conducted according to the principles of the declaration of Helsinki (version 2008).

10.2 Recruitment and consent
Patients with secondary adrenal insufficiency are recruited from the Endocrine outpatient clinic at the UMCG. The investigator will inform patients with secondary pituitary insufficiency about the study and ask for their consent after approval of their treating physician who will assess appropriateness of eligibility. The information will be by letter sent either by mail or handed out during a scheduled outpatient visit. It will be explained that participation is voluntary and refusal has no influence on the treatment of the patient. Any questions will be answered by the investigator. The subjects will be asked to react within two weeks if they want to participate by sending back a reaction form or by calling the investigator.

10.3 Objection by minors or incapacitated subjects (if applicable)
No incapacitated subjects or minors will be asked to participate.

10.4 Benefits and risks assessment, group relatedness
Benefit: participant's potential preference to either dosing scheme in addition to insight in risks and benefit of high or low dose HC treatment.
Risks: All HC dosing schemes can be considered safe and are published in literature. Additional hydrocortisone dose escape medication to prevend hypocortisolism is allowed. The risk of hypocortisolism on study dose-regimens of HC is small and estimated to be similar to conventional treatment at the outpatient clinic.

10.5 Compensation for injury

The sponsor/ investigator has a liability insurance which is in accordance with article 7, subsection 6 of the WMO.

The sponsor (also) has an insurance which is in accordance with the legal requirements in the Netherlands (article 7 WMO and the Measure regarding Compulsory Insurance for Clinical Research in Humans of 23th June 2003). This insurance provides cover for damage to research subjects through injury or death caused by the study.
1. € 450.000,-- (i.e. four hundred and fifty thousand Euro) for death or injury for each subject who participates in the Research;
2. € 3.500.000,-- (i.e. three million five hundred thousand euro) for death or injury for all subjects who participate in the Research;
3. € 5.000.000,-- (i.e. five million Euro) for the total damage incurred by the organization for all damage disclosed by scientific research for the Sponsor as 'verrichter' in the meaning of said Act in each year of insurance coverage.

The insurance applies to the damage that becomes apparent during the study or whitin 4 years after the end of the study.

10.6 Incentives (if applicable)
There will be no special incentives or compensations for subjects who participate. They only will be compensated for the travel and parking costs they made.

11 ADMINISTRATIVE ASPECTS AND PUBLICATION

11.1 Handling and storage of data and documents

Data will be handled confidentially and an identification code will be made. The codes are not based on the patients' initials or birth date. The key to the code will be safeguarded by the pharmacy of the UMCG. The handling of personal data will comply with the Dutch Personal Data Protection Act.

11.2 Amendments

Amendments are changes made to the research after a favourable opinion by the accredited METC has been given. All amendments will be notified to the METC that gave a favourable opinion.

11.3 Annual progress report

The sponsor/investigator will submit a summary of the progress of the trial to the accredited METC once a year. Information will be provided on the date of inclusion of the first subject, numbers of subjects included and numbers of subjects that have completed the trial, serious adverse events/ serious adverse reactions, other problems, and amendments.

11.4 End of study report

The investigator will notify the accredited METC of the end of the study within a period of 8 weeks. The end of the study is defined as the last patient's last visit.

In case the study is ended prematurely, the investigator will notify the accredited METC, including the reasons for the premature termination.

Within one year after the end of the study, the investigator/sponsor will submit a final study report with the results of the study, including any publications/abstracts of the study, to the accredited METC.

11.5 Public disclosure and publication policy

Publication policy will comply with the CCMO statement.

12 References

(1) Debono M, Price JN, Ross RJ. Novel strategies for hydrocortisone replacement. Best Pract Res Clin Endocrinol Metab 2009 Apr;23(2):221-32.

(2) Esteban NV, Loughlin T, Yergey AL, Zawadzki JK, Booth JD, Winterer JC, et al. Daily cortisol production rate in man determined by stable isotope dilution/mass spectrometry. J Clin Endocrinol Metab 1991 Jan;72(1):39-45.

(3) Kraan GP, Dullaart RP, Pratt JJ, Wolthers BG, Drayer NM, De Bruin R. The daily cortisol production reinvestigated in healthy men. The serum and urinary cortisol production rates are not significantly different. J Clin Endocrinol Metab 1998 Apr;83(4):1247-52.

(4) Nieman LK. Treatment of adrenal insufficiency in adults. [UptoDate]. 2011.
Ref Type: Generic

(5) Dullaart RP, van den Berg G, van der Knaap AM, Dijck-Brouwer J, Dallinga-Thie GM, Zelissen PM, et al. HDL cholesterol response to GH replacement is associated with common cholesteryl ester transfer protein gene variation (-629C>A) and modified by glucocorticoid treatment. Eur J Endocrinol 2010 Feb;162(2):227-34.

(6) Mah PM, Jenkins RC, Rostami-Hodjegan A, Newell-Price J, Doane A, Ibbotson V, et al. Weight-related dosing, timing and monitoring hydrocortisone replacement therapy in patients with adrenal insufficiency. Clin Endocrinol (Oxf) 2004 Sep;61(3):367-75.

(7) Arlt W, Rosenthal C, Hahner S, Allolio B. Quality of glucocorticoid replacement in adrenal insufficiency: clinical assessment vs. timed serum cortisol measurements. Clin Endocrinol (Oxf) 2006 Apr;64(4):384-9.

(8) Al Shoumer KA, Ali K, Anyaoku V, Niththyananthan R, Johnston DG. Overnight metabolic fuel deficiency in patients treated conventionally for hypopituitarism. Clin Endocrinol (Oxf) 1996 Aug;45(2):171-8.

(9) Filipsson H, Monson JP, Koltowska-Haggstrom M, Mattsson A, Johannsson G. The impact of glucocorticoid replacement regimens on metabolic outcome and comorbidity in hypopituitary patients. J Clin Endocrinol Metab 2006 Oct;91(10):3954-61.

(10) de Quervain DJ, Roozendaal B, Nitsch RM, McGaugh JL, Hock C. Acute cortisone administration impairs retrieval of long-term declarative memory in humans. Nat Neurosci 2000 Apr;3(4):313-4.

(11) Kirschbaum C, Wolf OT, May M, Wippich W, Hellhammer DH. Stress- and treatment-induced elevations of cortisol levels associated with impaired declarative memory in healthy adults. Life Sci 1996;58(17):1475-83.

(12) Lupien S, Lecours AR, Lussier I, Schwartz G, Nair NP, Meaney MJ. Basal cortisol levels and cognitive deficits in human aging. J Neurosci 1994 May;14(5 Pt 1):2893-903.

(13) Lupien SJ, Gaudreau S, Tchiteya BM, Maheu F, Sharma S, Nair NP, et al. Stress-induced declarative memory impairment in healthy elderly subjects: relationship to cortisol reactivity. J Clin Endocrinol Metab 1997 Jul;82(7):2070-5.

(14) Lupien SJ, Gillin CJ, Hauger RL. Working memory is more sensitive than declarative memory to the acute effects of corticosteroids: a dose-response study in humans. Behav Neurosci 1999 Jun;113(3):420-30.

(15) Lupien SJ, Wilkinson CW, Briere S, Menard C, Ng Ying Kin NM, Nair NP. The modulatory effects of corticosteroids on cognition: studies in young human populations. Psychoneuroendocrinology 2002 Apr;27(3):401-16.

(16) Maheu FS, Collicutt P, Kornik R, Moszkowski R, Lupien SJ. The perfect time to be stressed: a differential modulation of human memory by stress applied in the morning or in the afternoon. Prog Neuropsychopharmacol Biol Psychiatry 2005 Dec;29(8):1281-8.

(17) McCormick CM, Lewis E, Somley B, Kahan TA. Individual differences in cortisol levels and performance on a test of executive function in men and women. Physiol Behav 2007 May 16;91(1):87-94.

(18) Newcomer JW, Craft S, Hershey T, Askins K, Bardgett ME. Glucocorticoid-induced impairment in declarative memory performance in adult humans. J Neurosci 1994 Apr;14(4):2047-53.

(19) Newcomer JW, Selke G, Melson AK, Hershey T, Craft S, Richards K, et al. Decreased memory performance in healthy humans induced by stress-level cortisol treatment. Arch Gen Psychiatry 1999 Jun;56(6):527-33.

(20) Young AH, Sahakian BJ, Robbins TW, Cowen PJ. The effects of chronic administration of hydrocortisone on cognitive function in normal male volunteers. Psychopharmacology (Berl) 1999 Aug;145(3):260-6.

(21) Lupien SJ, Wilkinson CW, Briere S, Ng Ying Kin NM, Meaney MJ, Nair NP. Acute modulation of aged human memory by pharmacological manipulation of glucocorticoids. J Clin Endocrinol Metab 2002 Aug;87(8):3798-807.

(22) Lupien SJ, Fiocco A, Wan N, Maheu F, Lord C, Schramek T, et al. Stress hormones and human memory function across the lifespan. Psychoneuroendocrinology 2005 Apr;30(3):225-42.

(23) Wolkowitz OM, Reus VI, Weingartner H, Thompson K, Breier A, Doran A, et al. Cognitive effects of corticosteroids. Am J Psychiatry 1990 Oct;147(10):1297-303.

(24) McGaugh JL, McIntyre CK, Power AE. Amygdala modulation of memory consolidation: interaction with other brain systems. Neurobiol Learn Mem 2002 Nov;78(3):539-52.

(25) Maheu FS, Collicutt P, Kornik R, Moszkowski R, Lupien SJ. The perfect time to be stressed: a differential modulation of human memory by stress applied in the morning or in the afternoon. Prog Neuropsychopharmacol Biol Psychiatry 2005 Dec;29(8):1281-8.

(26) Roozendaal B. Stress and memory: opposing effects of glucocorticoids on memory consolidation and memory retrieval. Neurobiol Learn Mem 2002 Nov;78(3):578-95.

(27) Lupien SJ, Lepage M. Stress, memory, and the hippocampus: can't live with it, can't live without it. Behav Brain Res 2001 Dec 14;127(1-2):137-58.

(28) Kim JJ, Diamond DM. The stressed hippocampus, synaptic plasticity and lost memories. Nat Rev Neurosci 2002 Jun;3(6):453-62.

(29) Abe K. Modulation of hippocampal long-term potentiation by the amygdala: a synaptic mechanism linking emotion and memory. Jpn J Pharmacol 2001 May;86(1):18-22.

(30) Mizoguchi K, Ishige A, Takeda S, Aburada M, Tabira T. Endogenous glucocorticoids are essential for maintaining prefrontal cortical cognitive function. J Neurosci 2004 Jun 16;24(24):5492-9.

(31) Sanchez MM, Young LJ, Plotsky PM, Insel TR. Distribution of corticosteroid receptors in the rhesus brain: relative absence of glucocorticoid receptors in the hippocampal formation. J Neurosci 2000 Jun 15;20(12):4657-68.

(32) Cerqueira JJ, Almeida OF, Sousa N. The stressed prefrontal cortex. Left? Right! Brain Behav Immun 2008 Jul;22(5):630-8.

(33) Rolke R, Magerl W, Campbell KA, Schalber C, Caspari S, Birklein F, et al. Quantitative sensory testing: a comprehensive protocol for clinical trials. Eur J Pain 2006 Jan;10(1):77-88.

(34) Kuehl LK, Michaux GP, Richter S, Schachinger H, Anton F. Increased basal mechanical pain sensitivity but decreased perceptual wind-up in a human model of relative hypocortisolism. Pain 2010 Jun;149(3):539-46.

13 Appendix

13.1 Appendix 1

Cognitive test battery

Somatic complaints
The Daily mood and symptom report

Attention
Test of Attentional Performance (TAP)

Memory
15 Words Test (15 WT)
Rivermead Behavioral Memory Test (RBMT)
Digit span forward (short term memory)
Rey 15 figures Test
Figure of Rey and Osterrieth

Executive functioning
Digit span backward
Verbal fluency:
- phonetic (d,a,t; k,o,m; p,g,r)
- semantic (animals; professions; supermarket articles)

Trail Making Test A/B

Social Cognition
Reading the mind in the eyes test (RMET)
Go/No-go task (neutral vs. emotional)

13.2 Appendix 2

The Daily mood and symptom report

Hydrocortison substitutie bij patiënten met secundaire bijnierschorsinsufficiëntie

Dagboeken van studie week ..

Patient nummer (In te vullen door de onderzoeker):

Datum:

	**Hoeveel last hebt u in de afgelopen 24 uur gehad van één of meer van de volgende problemen ?**	**Geen last**						**Veel last**
1.	Buikpijn	1	2	3	4	5	6	7
2.	Rugpijn	1	2	3	4	5	6	7
3.	Pijn in uw armen, benen of							
	gewrichten (knieën, heupen, enz.)	1	2	3	4	5	6	7
4.	Menstruatiepijn of andere							
	problemen tijdens de menstruatie	1	2	3	4	5	6	7
5.	Pijn of problemen bij seksuele							
	gemeenschap	1	2	3	4	5	6	7
6.	Hoofdpijn							
7.	Pijn in de borstkas	1	2	3	4	5	6	7
8.	Duizeligheid							
9.	Flauwvallen	1	2	3	4	5	6	7
10.	Uw hart voelen bonzen of snel	1	2	3	4	5	6	7
	kloppen	1	2	3	4	5	6	7
11.	Kortademigheid	1	2	3	4	5	6	7
12.	Verstopping (obstipatie), dunne	1	2	3	4	5	6	7
	ontlasting of diarree							
13.	Misselijkheid, opgeblazen gevoel	1	2	3	4	5	6	7
	of problemen met de spijsvertering	1	2	3	4	5	6	7
	(indigestie)							

Datum:

**Hoeveel last hebt u in de afgelopen 24 uur gehad van één of meer van de volgende problemen?**	**Geen last**						**Veel last**
1. U nerveus, angstig of gespannen voelen	1	2	3	4	5	6	7
2. Niet kunnen stoppen met u zorgen maken beteugelen	1	2	3	4	5	6	7
3. U te veel zorgen maken om verschillende dingen	1	2	3	4	5	6	7
4. Moeite hebben u te ontspannen	1	2	3	4	5	6	7
5. Zo rusteloos zijn dat het moeilijk is om stil te zitten	1	2	3	4	5	6	7
6. Snel geërgerd of geïrriteerd zijn	1	2	3	4	5	6	7
7. Bang zijn dat er iets vreselijks zou kunnen gebeuren	1	2	3	4	5	6	7

Datum:

**Hoeveel last hebt u in de afgelopen 24 uur gehad van één of meer van de volgende problemen ?**	**Geen last**						**Veel last**
1. Weinig interesse of plezier in activiteiten	1	2	3	4	5	6	7
2. Zich neerslachtig, depressief of hopeloos voelen	1	2	3	4	5	6	7
3. Moeilijk inslapen, moeilijk doorslapen of te veel slapen	1	2	3	4	5	6	7
4. Zich moe voelen of gebrek aan energie hebben	1	2	3	4	5	6	7
5. Weinig eetlust of overmatig eten	1	2	3	4	5	6	7
6. Een slecht gevoel hebben over uzelf – of het gevoel hebben dat u een mislukkeling bent of het gevoel dat u zichzelf of uw familie teleurgesteld hebt	1	2	3	4	5	6	7
7. Problemen om u te concentreren, bijvoorbeeld om de krant te lezen of om tv te kijken	1	2	3	4	5	6	7
8. Zo traag bewegen of zo langzaam spreken dat andere mensen dit opgemerkt kunnen hebben? Of het tegenovergestelde, zo zenuwachtig of rusteloos zijn dat u veel meer bewoog dan gebruikelijk	1	2	3	4	5	6	7
9. De gedachte dat u beter dood zou kunnen zijn of de gedachte uzelf op een bepaalde manier pijn te doen	1	2	3	4	5	6	7

Als u enig probleem hebt aangekruist, hoe moeilijk maakten deze problemen het dan voor u om uw werk of uw taken in en om het huis te doen, of om andere mensen om te gaan?

Helemaal niet moeilijk						Extreem moeilijk
**1**	**2**	**3**	**4**	**5**	**6**	**7**

Denk aan de voor u belangrijkste gebeurtenis van de afgelopen 24 uur.

Hoe plezierig was deze gebeurtenis?

Zeer onplezierig Zeer plezierig

☐ -3 ☐ -2 ☐ -1 ☐ 0 ☐ 1 ☐ 2 ☐ 3

Dit was belangrijk voor mij

Zeer onbelangrijk Zeer belangrijk

☐ -3 ☐ -2 ☐ -1 ☐ 0 ☐ 1 ☐ 2 ☐ 3

Ik had dit onder controle

Niet Matig Zeer

☐ 1 ☐ 2 ☐ 3 ☐ 4 ☐ 5 ☐ 6 ☐ 7

Was deze situatie onverwacht?

Niet Matig Zeer

☐ 1 ☐ 2 ☐ 3 ☐ 4 ☐ 5 ☐ 6 ☐ 7

Bent u vandaag één/ enkele dosering(en) vergeten?

☐ Ja, namelijk voor het ontbijt / voor de lunch / voor het avondeten (doorstrepen wat niet van toepassing is)

☐ Nee

Heeft u vandaag één/ enkele dosering(en) verdubbeld ?

☐ Ja, namelijk voor het ontbijt / voor de lunch / voor het avondeten (doorstrepen wat niet van toepassing is)

☐ Nee

13.3 Appendix 3
Instructions for the questionnaires

1.
Instructies voor het invullen van de vragenlijst

Wilt u voordat u begint met het beantwoorden van de vragen de volgende punten doorlezen?

1. Deze vragenlijst bestaat uit meerdere onderdelen. Elk onderdeel begint met een inleiding, waarin staat wat de bedoeling is van de vragen en hoe u deze moet beantwoorden.

2. De vragen kunt u beantwoorden door het juiste antwoord aan te kruisen of te omcirkelen. Geef slechts één antwoord!

3. Denk niet te lang na per vraag; uw eerste ingeving is vaak de beste.

4. Het is belangrijk dat u uw eigen mening geeft en de vragen zonder overleg met anderen invult. Er zijn geen goede of foute antwoorden.

5. Tijdens het invullen van de vragenlijst zult u merken dat veel vragen op elkaar lijken. Dit komt omdat deze vragenlijst is samengesteld uit verschillende reeds bestaande vragenlijsten. Het is belangrijk om alle vragen toch in te vullen, ondanks dat u het gevoel heeft een paar keer hetzelfde in te vullen.

6. Als u alle vragen heeft ingevuld, kunt u deze vragenlijst samen met uw toestemmingsverklaring terugsturen in de antwoordenvelop.

7. Wanneer u problemen tegenkomt bij het invullen van de vragenlijst of vragen heeft over het onderzoek, dan kunt u contact opnemen met Pauline Brummelman.

Heel veel succes met het invullen van de vragenlijst en hartelijk dank voor uw medewerking!
Dit levert ons zeer waardevolle informatie op!

Pauline Brummelman
onderzoekster afdeling endocrinologie UMCG

Telefoonnummer: 050 -3612978
(bij geen gehoor kunt u een bericht inspreken, dan bel ik u zo spoedig mogelijk terug)
Eventueel kunt u bellen met het secretariaat endocrinologie (050 3613962) en daar een bericht achterlaten.

Emailadres: p.brummelman@umcg.nl

Daily activities / Dagelijkse werkzaamheden

Hieronder volgen enkele vragen over uw dagelijkse bezigheden. Kruis het hokje aan dat op u van toepassing is.

1. Doet u op dit moment betaald werk?
(indien u in de ziektewet zit, deze vraag ook met 'ja' beantwoorden)

☐ Ja
☐ Nee (ga door naar vraag 5)

2. Hoeveel uren werkt u gewoonlijk in de week? Indien u onregelmatig werkt, graag een schatting geven van het gemiddelde aantal uren per week.
(deze vraag ook beantwoorden als u in de ziektewet zit)

.... uur per week

3. Zit u momenteel in de ziektewet?

☐ Ja
☐ Nee (ga door naar vraag 5)

4. Indien u momenteel in de ziektewet zit, werkt u dan:

☐ Helemaal niet
☐ Gedeeltelijk, namelijk .... uur per week

5. Kunt u aangeven tot welke van de onderstaande groepen u behoort? Wilt u aankruisen wat in de eerste plaats op u van toepassing is? Niet meer dan één mogelijkheid aankruisen alstublieft.

☐ Ik ben fulltime / parttime werkend
☐ Ik zoek werk na verlies van mijn vorige baan
☐ Ik zoek voor het eerst werk / zoek werk na langdurige onderbreking
☐ Ik ben scholier / student
☐ Ik doe het huishouden
☐ Ik ben gepensioneerd
☐ Ik ben met vervroegd pensioen (VUT)
☐ Ik ben geheel of gedeeltelijk arbeidsongeschikt
☐ Ik doe onbetaald werk met behoud van uitkering / doe vrijwilligerswerk
☐ Ik doe iets anders, namelijk...

Health (RAND-36) / Gezondheid (RAND-36)

In deze vragenlijst wordt naar uw gezondheid gevraagd. Wilt u elke vraag beantwoorden door het juiste hokje aan te kruisen? Wanneer u twijfelt over het antwoord op een vraag, probeer dan het antwoord te geven dat het meest van toepassing is.

1. Wat vindt u, over het algemeen genomen, van uw gezondheid?

☐ Uitstekend
☐ Zeer goed
☐ Goed
☐ Matig

1.1.1 ☐ Slecht

2. *In vergelijking met een jaar geleden*, hoe zou u *nu* uw gezondheid in het algemeen beoordelen?

☐ Veel beter dan een jaar geleden
☐ Iets beter dan een jaar geleden
☐ Ongeveer hetzelfde als een jaar geleden
☐ Iets slechter dan een jaar geleden
☐ Veel slechter dan een jaar geleden

3. De volgende vragen gaan over dagelijkse bezigheden. Wordt u door uw gezondheid *op dit moment* beperkt bij deze bezigheden? Zo ja, in welke mate?

		**Ja, ernstig beperkt**	**Ja, een beetje beperkt**	**Nee, helemaal niet beperkt**
a	Forse inspanning<br>zoals hardlopen, zware voorwerpen tillen, inspannend sporten	☐	☐	☐
b	Matige inspanning<br>zoals het verplaatsen van een tafel, stofzuigen, fietsen	☐	☐	☐
c	Tillen of boodschappen dragen	☐	☐	☐
d	*Een paar* trappen oplopen	☐	☐	☐
e	*Eén* trap oplopen	☐	☐	☐
f	Buigen, knielen of bukken	☐	☐	☐
g	*Meer dan een kilometer* lopen	☐	☐	☐
h	*Een halve kilometer* lopen	☐	☐	☐

i	*Honderd meter* lopen	□	□	□
j	**1.2 Uzelf wassen of aankleden**	□	□	□

1.3

4. Had u, ten gevolge van uw lichamelijke gezondheid, *de afgelopen 4 weken* één van de volgende problemen bij uw werk of andere dagelijkse bezigheden?

		) ***Ja***	) ***Nee***
a	U heeft *minder tijd* kunnen besteden aan werk of andere bezigheden	□	□
b	U heeft *minder bereikt* dan u zou willen	□	□
c	U was beperkt in het *soort* werk of het soort bezigheden	□	□
d	U had moeite met het werk of andere bezigheden (het kostte u bijvoorbeeld extra inspanning)	□	□

5. Had u, ten gevolge van een emotioneel probleem (bijvoorbeeld doordat u zich depressief of angstig voelde), *de afgelopen 4 weken* één van de volgende problemen bij uw werk of andere dagelijkse bezigheden?

		) ***Ja***	) ***Nee***
a	U heeft *minder tijd* kunnen besteden aan werk of andere bezigheden	□	□
b	U heeft *minder bereikt* dan u zou willen	□	□
c	U heeft het werk of andere bezigheden niet zo zorgvuldig gedaan als u gewend bent	□	□

6. In hoeverre heeft uw lichamelijke gezondheid of hebben uw emotionele problemen u *de afgelopen 4 weken* belemmerd in uw normale sociale bezigheden met gezin, vrienden, buren of anderen?

□ Helemaal niet
□ Enigszins
□ Nogal
□ Veel
□ Heel erg veel

7. Hoeveel pijn had u *de afgelopen vier weken*?

☐ Geen
☐ Heel licht
☐ Licht
☐ Nogal
☐ Ernstig
☐ Heel ernstig

8. In welke mate heeft pijn u *de afgelopen 4 weken* belemmerd bij uw normale werkzaamheden (zowel werk buitenshuis als huishoudelijk werk)?

☐ Helemaal niet
☐ Een klein beetje
☐ Nogal
☐ Veel
☐ Heel erg veel

9. Deze vragen gaan over hoe u zich *de afgelopen 4 weken* heeft gevoeld. Wilt u bij elke vraag het antwoord aankruisen dat het beste aansluit bij hoe u zich heeft gevoeld?

Hoe vaak gedurende *de afgelopen vier weken*:

		**Voortdurend**	**Meestal**	**Vaak**	**Soms**	**Zelden**	**Nooit**
a	voelde u zich levenslustig?	☐	☐	☐	☐	☐	☐
b	voelde u zich erg zenuwachtig?	☐	☐	☐	☐	☐	☐
c	zat u zo erg in de put dat niets u kon opvrolijken?	☐	☐	☐	☐	☐	☐
d	voelde u zich kalm en rustig?	☐	☐	☐	☐	☐	☐
e	voelde u zich erg energiek?	☐	☐	☐	☐	☐	☐
f	voelde u zich neerslachtig en somber?	☐	☐	☐	☐	☐	☐
g	voelde u zich uitgeblust?	☐	☐	☐	☐	☐	☐
h	voelde u zich gelukkig?	☐	☐	☐	☐	☐	☐
i	voelde u zich moe?	☐	☐	☐	☐	☐	☐

10. *Hoe vaak* hebben uw lichamelijke gezondheid of emotionele problemen gedurende *de afgelopen 4 weken* uw sociale activiteiten (zoals bezoek aan vrienden of naaste familieleden) belemmerd?

☐ Voortdurend
☐ Meestal
☐ Soms
☐ Zelden
☐ Nooit

11. Wilt u het antwoord kiezen dat het beste weergeeft hoe juist of onjuist u elk van de volgende uitspraken voor uzelf vindt?

		**Volkomen juist**	**Grotendeels juist**	**Weet ik niet**	**Grotendeels onjuist**	**Volkomen onjuist**
a	Ik lijk gemakkelijker ziek te worden dan andere mensen	☐	☐	☐	☐	☐
b	Ik ben net zo gezond als andere mensen die ik ken	☐	☐	☐	☐	☐
c	Ik verwacht dat mijn gezondheid achteruit zal gaan	☐	☐	☐	☐	☐
d	Mijn gezondheid is uitstekend	☐	☐	☐	☐	☐

Welfare (HADS) / Welzijn (HADS)

Wij willen graag weten hoe u zich de laatste tijd heeft gevoeld. Wilt u bij elke vraag het cijfer vóór het antwoord dat het meest op u van toepassing is omcirkelen? Denk erom, het gaat bij deze vragen om hoe u zich de laatste tijd (in het bijzonder de afgelopen 4 weken) voelde, dus niet om hoe u zich in het verleden heeft gevoeld.

1.	Ik voel me de laatste tijd gespannen.	0	Meestal
		1	Vaak
		2	Af en toe, soms
		3	Helemaal niet
2.	Ik geniet nog steeds van de dingen waar ik vroeger van genoot.	0	Zeker zo veel
		1	Niet helemaal zoveel
		2	Weinig
		3	Eigenlijk helemaal niet
3.	Ik krijg de laatste tijd het angstige gevoel alsof er elk moment iets vreselijks zal gebeuren.	0	Heel zeker en vrij erg
		1	Ja, maar niet zo erg
		2	Een beetje, maar ik maak me er geen zorgen over
		3	Helemaal niet
4.	Ik kan lachen en de dingen van de vrolijke kant zien.	0	Net zoveel als vroeger
		1	Niet zo goed meer nu
		2	Beslist niet zoveel als vroeger
		3	Helemaal niet
5.	Ik maak me de laatste tijd ongerust.	0	Heel erg vaak
		1	Vaak
		2	Af en toe
		3	Zelden of nooit
6.	Ik voel me de laatste tijd opgewekt.	0	Helemaal niet
		1	Niet vaak
		2	Soms
		3	Meestal
7.	Ik kan de laatste tijd rustig zitten en me ontspannen.	0	Zeker
		1	Meestal
		2	Niet vaak
		3	Helemaal niet
8.	Ik voel me de laatste tijd alsof alles moeizamer gaat.	0	Bijna altijd
		1	Heel vaak
		2	Soms
		3	Helemaal niet
9.	Ik krijg de laatste tijd een soort benauwd,	0	Helemaal niet

	gespannen gevoel in mijn maag.	1	Soms
		2	Vrij vaak
		3	Heel vaak
10.	Ik heb de laatste tijd geen interesse meer in mijn uiterlijk.	0	Zeker
		1	Niet meer zoveel als ik zou moeten
		2	Mogelijk wat minder
		3	Evenveel interesse als voorheen
11.	Ik voel me de laatste tijd rusteloos.	0	Heel erg
		1	Tamelijk veel
		2	Niet erg veel
		3	Helemaal niet
12.	Ik verheug me van tevoren al op dingen.	0	Net zoveel als vroeger
		1	Een beetje minder dan vroeger
		2	Zeker minder dan vroeger
		3	Bijna nooit
13.	Ik krijg de laatste tijd plotseling gevoelens van angst of paniek.	0	Zeer vaak
		1	Tamelijk vaak
		2	Niet erg vaak
		3	Helemaal niet
14.	Ik kan van een goed boek genieten, of van een radio - of televisieprogramma.	0	Vaak
		1	Soms
		2	Niet vaak
		3	Heel zelden

Fatigue (MVI-20)/ Vermoeidheid (MVI-20)

Met behulp van onderstaande uitspraken willen wij een indruk krijgen van hoe u zich *de laatste dagen* voelt.

Voorbeeld:

“ Ik voel me ontspannen.”

Wanneer u vindt dat het *helemaal* klopt dat u zich *de laatste dagen* ontspannen voelt, plaatst u een kruisje in het linker hokje; dus zo:

ja, dat klopt	☐	☐	☐	☐	☐	nee, dat klopt niet

Hoe minder u de uitspraak van toepassing vindt, hoe meer u het kruisje naar rechts richting 'nee, dat klopt niet ' kunt plaatsen. Sla geen uitspraken over en plaats telkens één kruisje bij iedere uitspraak. Er zijn geen foute antwoorden. Het gaat om uw eerste indruk.

1.	Ik voel me fit.	ja, dat klopt	☐	☐	☐	☐	☐	nee, dat klopt niet
2.	Lichamelijk voel ik me tot weinig in staat.	ja, dat klopt	☐	☐	☐	☐	☐	nee, dat klopt niet
3.	Ik zit vol activiteit.	ja, dat klopt	☐	☐	☐	☐	☐	nee, dat klopt niet
4.	Ik heb zin om allerlei leuke dingen te gaan doen.	ja, dat klopt	☐	☐	☐	☐	☐	nee, dat klopt niet
5.	Ik voel me moe.	ja, dat klopt	☐	☐	☐	☐	☐	nee, dat klopt niet
6.	Ik vind dat ik veel doe op een dag.	ja, dat klopt	☐	☐	☐	☐	☐	nee, dat klopt niet
7.	Als ik ergens mee bezig ben, kan ik mijn gedachten er goed bij houden.	ja, dat klopt	☐	☐	☐	☐	☐	nee, dat klopt niet
8.	Lichamelijk kan ik veel aan.	ja, dat klopt	☐	☐	☐	☐	☐	nee, dat klopt niet
9.	Ik zie er tegen op om iets te doen.	ja, dat klopt	☐	☐	☐	☐	☐	nee, dat klopt niet
10.	Ik vind dat ik weinig doe op een dag.	ja, dat klopt	☐	☐	☐	☐	☐	nee, dat klopt niet
11.	Ik kan me goed concentreren.	ja, dat klopt	☐	☐	☐	☐	☐	nee, dat klopt niet
12.	Ik voel me uitgerust.	ja, dat	☐	☐	☐	☐	☐	nee, dat

		klopt	☐	☐	☐	☐	☐	klopt niet
13.	Het kost me moeite ergens mijn aandacht bij te houden.	ja, dat klopt	☐	☐	☐	☐	☐	nee, dat klopt niet
14.	Lichamelijk voel ik me in een slechte conditie.	ja, dat klopt	☐	☐	☐	☐	☐	nee, dat klopt niet
15.	Ik zit vol plannen.	ja, dat klopt	☐	☐	☐	☐	☐	nee, dat klopt niet
16.	Ik ben gauw moe.	ja, dat klopt	☐	☐	☐	☐	☐	nee, dat klopt niet
17.	Er komt weinig uit mijn handen.	ja, dat klopt	☐	☐	☐	☐	☐	nee, dat klopt niet
18.	De zin om dingen te ondernemen ontbreekt mij.	ja, dat klopt	☐	☐	☐	☐	☐	nee, dat klopt niet
19.	Mijn gedachten dwalen makkelijk af.	ja, dat klopt	☐	☐	☐	☐	☐	nee, dat klopt niet
20.	Lichamelijk voel ik me in een uitstekende conditie.	ja, dat klopt	☐	☐	☐	☐	☐	nee, dat klopt niet

Cognition (CFQ) / Cognitie (CFQ)

De volgende 25 vragen gaan over kleine, alledaagse vergissingen die iedereen van tijd tot tijd maakt. Hieronder kunt u steeds het hokje aankruisen dat bij uw antwoord hoort. Per vraag slechts één hokje aankruisen.

Voorbeeld:	Zeer vaak	Vaak	Af en toe	Zelden	Nooit
Uw bril zoeken die op u voorhoofd zit.	□	□	□	■	□

Stel, u bent maar zelden uw bril kwijt op die manier. U kruist dan het hokje 'zelden' aan, zoals hierboven. Als u deze vergissing zeer vaak maakt, dan kruist u het hokje 'zeer vaak' aan. Als het u nooit overkomt, kruist u het hokje 'nooit' aan.

		Zeer vaak	Vaak	Af en toe	Zelden	Nooit
1.	Iets lezen en vlak daarna niet meer weten wat u nu gelezen heeft, zodat u het moet overlezen.	□	□	□	□	□
2.	Vergeten waarom u naar een bepaald gedeelte van uw huis bent gelopen.	□	□	□	□	□
3.	Wegwijzers over het hoofd zien.	□	□	□	□	□
4.	Links en rechts verwarren bij het beschrijven van een route.	□	□	□	□	□
5.	Per ongeluk tegen mensen opbotsen.	□	□	□	□	□
6.	Niet meer weten of u het licht of gas hebt uitgedaan, of de deur hebt afgesloten.	□	□	□	□	□

		Zeer vaak	Vaak	Af en toe	Zelden	Nooit
7.	Niet luisteren naar de naam van een persoon op het moment dat deze persoon zich aan u voorstelt.	□	□	□	□	□
8.	Iets eruit flappen en achteraf bedenken dat dat wel eens beledigend voor iemand zou kunnen zijn.	□	□	□	□	□
9.	Niet merken dat iemand iets tegen u zegt als u met iets anders bezig bent.	□	□	□	□	□
10.	Boos worden en daar later spijt van hebben.	□	□	□	□	□
11.	Belangrijke brieven dagenlang onbeantwoord laten.	□	□	□	□	□
12.	Vergeten welke straat u moet inslaan als u een route kiest die u goed kent, maar die u	□	□	□	□	□

	maar zelden gebruikt.					
13.	In een supermarkt niet kunnen vinden wat u zoekt, terwijl het er wél is.	☐	☐	☐	☐	☐
14.	U plotseling afvragen of u een woord op de juiste manier gebruikt.	☐	☐	☐	☐	☐
15.	Moeite hebben met het nemen van een beslissing.	☐	☐	☐	☐	☐
16.	Afspraken vergeten.	☐	☐	☐	☐	☐
17.	Vergeten waar u iets hebt neergelegd, zoals een boek of een krant.	☐	☐	☐	☐	☐
18.	Per ongeluk iets weggooien dat u nodig hebt en bewaren wat u weg wilde gooien.	☐	☐	☐	☐	☐
19.	Dagdromen terwijl u eigenlijk naar iets of iemand zou moeten luisteren.	☐	☐	☐	☐	☐
20.	Namen van mensen vergeten.	☐	☐	☐	☐	☐
21.	Beginnen met iets, maar het niet afmaken omdat u ongemerkt met iets anders bent begonnen.	☐	☐	☐	☐	☐
22.	Niet op een woord kunnen komen terwijl het ‘op het puntje van uw tong’ ligt.	☐	☐	☐	☐	☐
23.	In een winkel vergeten wat u er kwam kopen.	☐	☐	☐	☐	☐
24.	Dingen uit uw handen laten vallen.	☐	☐	☐	☐	☐
		**Zeer vaak**	**Vaak**	**Af en toe**	**Zelden**	**Nooit**
25.	In een gesprek niets meer weten om over te praten.	☐	☐	☐	☐	☐

Als u bij *alle* 25 vragen over alledaagse vergissingen op deze en de vorige bladzijden het hokje ‘nooit’ heeft aangekruist, dan kunt u onderstaande vragen overslaan.

26. Is het maken van deze alledaagse vergissingen in de afgelopen 5 jaar bij u toegenomen?

☐ Helemaal niet toegenomen
☐ Een klein beetje toegenomen
☐ Matig toegenomen
☐ Nogal sterk toegenomen
☐ Zeer sterk toegenomen

27. Hoeveel hinder heeft u van het maken van deze vergissingen in het dagelijks leven?

☐ Helemaal geen hinder
☐ Zeer weinig hinder
☐ Een beetje hinder
☐ Veel hinder
☐ Zeer veel hinder

28. In hoeverre maakt u zich zorgen over het maken van deze vergissingen in het dagelijks leven?

☐ Helemaal geen zorgen
☐ Zeer weinig zorgen
☐ Een beetje zorgen
☐ Veel zorgen
☐ Zeer veel zorgen

29. Kunt u aangeven in hoeverre u zich ergert aan het maken van deze alledaagse vergissingen?

☐ Het ergert mij helemaal niet
☐ Het ergert mij een beetje
☐ Het ergert mij matig
☐ Het ergert mij nogal veel
☐ Het ergert mij zeer veel

Personal Information / Persoonlijke gegevens

De volgende vragen betreffen uw persoonlijke gegevens. Kruis het antwoord aan dat op u van toepassing is of vul het antwoord in op de stippellijn.

1. Wat is uw geboortejaar?

   19.......

2. Wat is uw geslacht?

- ☐ Man
- ☐ Vrouw

3. Wat is uw burgerlijke staat?

- ☐ Gehuwd
- ☐ Samenwonend
- ☐ Partner, niet samenwonend
- ☐ Ongehuwd
- ☐ Gescheiden
- ☐ Weduwe / weduwnaar

4. Wat is de duur van uw huidige relatie?

   ....

5. Hoeveel kinderen heeft u?

   .... kinderen

6. Hoeveel van uw kinderen zijn nog thuiswonend?

   .... kinderen

7. Wat is uw hoogst genoten opleiding?

- ☐ Lager onderwijs / basisonderwijs
- ☐ VGLO (voortgezet lager onderwijs)
- ☐ LBO (lager beroepsonderwijs)
- ☐ ULO, MULO, 3-jarige HBS of MAVO
- ☐ MBO (middelbaar beroepsonderwijs, bijvoorbeeld MEAO, MTS)
- ☐ 5-jarige HBS, HAVO, MMS, atheneum, gymnasium
- ☐ HBO (hoger beroepsonderwijs)
- ☐ Universiteit
- ☐ Anders, namelijk...

8. Heeft u last van één van de volgende aandoeningen? Er zijn meerdere antwoorden mogelijk.
....
....
....
....
....
....
....

HARTELIJK DANK VOOR UW MEDEWERKING!

Appendix 4 Somatosensation / Gevoelswaarneming

Quantitative Sensory Testing (QST)

Mechanical QST tests consist of seven different parameters. The mechanical detection threshold (MDT) is determined by using a standardized set of modified von Frey filaments (Optihair2-Set, Marstock Nervtest, Germany). MDT is assessed by using the ‘‘method of limits’’ in which five threshold determinations are made, each with a series of ascending and descending stimulus intensities. The final threshold is the geometric mean of these five series.

The mechanical pain threshold (MPT) is measured using a custom made set of seven pinprick devices with fixed stimulus intensities that exerted forces of 8, 16, 32, 64, 128, 256, and 512 mN. The stimulators are applied in an ascending order until the first percept of sharpness is reached. The final threshold is the geometric mean of five series of ascending and descending stimuli.

Mechanical pain sensitivity (MPS) is assessed using the same set of seven weighted pinprick stimuli to obtain a stimulus–response function for pinprick-evoked pain. Subjects are asked to give a pain rating for each stimulus on a ‘0–100’ numerical rating scale (NRS) (‘0’ indicating ‘‘no pain’’, and ‘100’ indicating ‘‘most intense pain imaginable’’).

Dynamic mechanical allodynia (ALL) was assessed as part of the test above, using a light tactile stimulator as moving innocuous stimuli: a standardized brush (Somedic, Sweden). A total of 40 stimuli, 5 tactile and 35 pinprick, are delivered in balanced and pseudo-randomized order at each site with the subject giving numerical pain ratings for each stimulus. MPS and ALL are calculated as the geometric mean of all numerical ratings for pinprick stimuli and all light touch stimulators, respectively.

Wind up ratio (WUR) test is defined as the NRS score ratio of a single pinprick stimulus (256mN) vs. the NRS score of a train of 10 pinprick stimuli of the same force. The pressure pain threshold (PPT) is determined over muscle with a pressure gauge device (FDN200, Wagner Instruments, USA) with three series of ascending stimulus intensities, each applied as a slowly increasing ramp of 50 kPa/s (0.5 kg/cm2 s). The final threshold is the geometric mean of three stimulations, each.
